# Supplementary material for: The Role of Apoptosis and Autophagy in the Hypothalamic-Pituitary-Adrenal (HPA) Axis after Traumatic Brain Injury (TBI)
Source: Int J Mol Sci. 2022 Dec 10;23(24):15699. doi: 10.3390/ijms232415699 (PMC9778890; doi:10.3390/ijms232415699)
Supplement: Supplementary file 1 [file ijms-23-15699-s001.zip › ijms-2042873-supplementary.pdf]

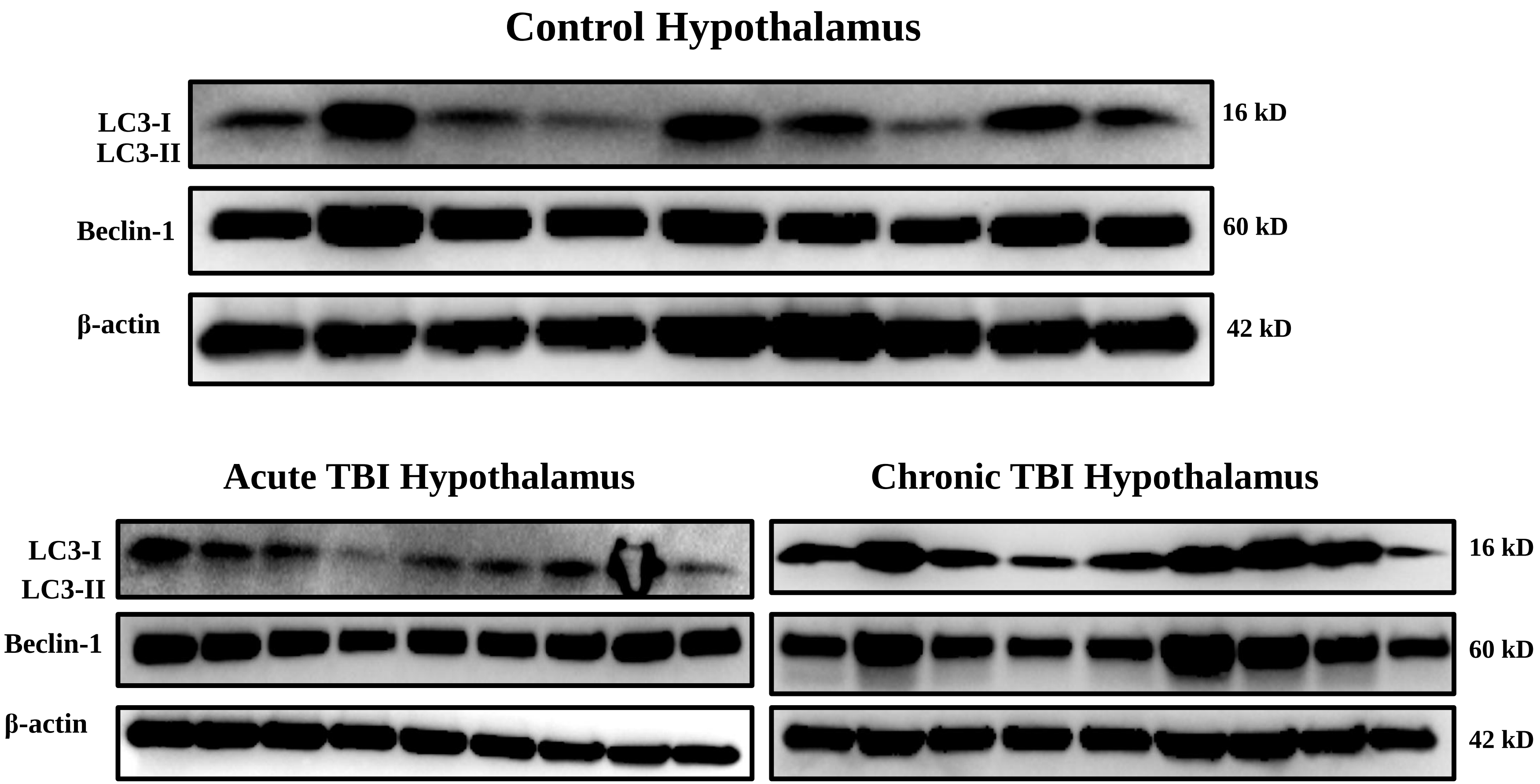

Supp. Figure S1. A. Western blot images of LC3-I and Beclin-1 in the hypothalamus after TBI in the acute, chronic, and control groups.

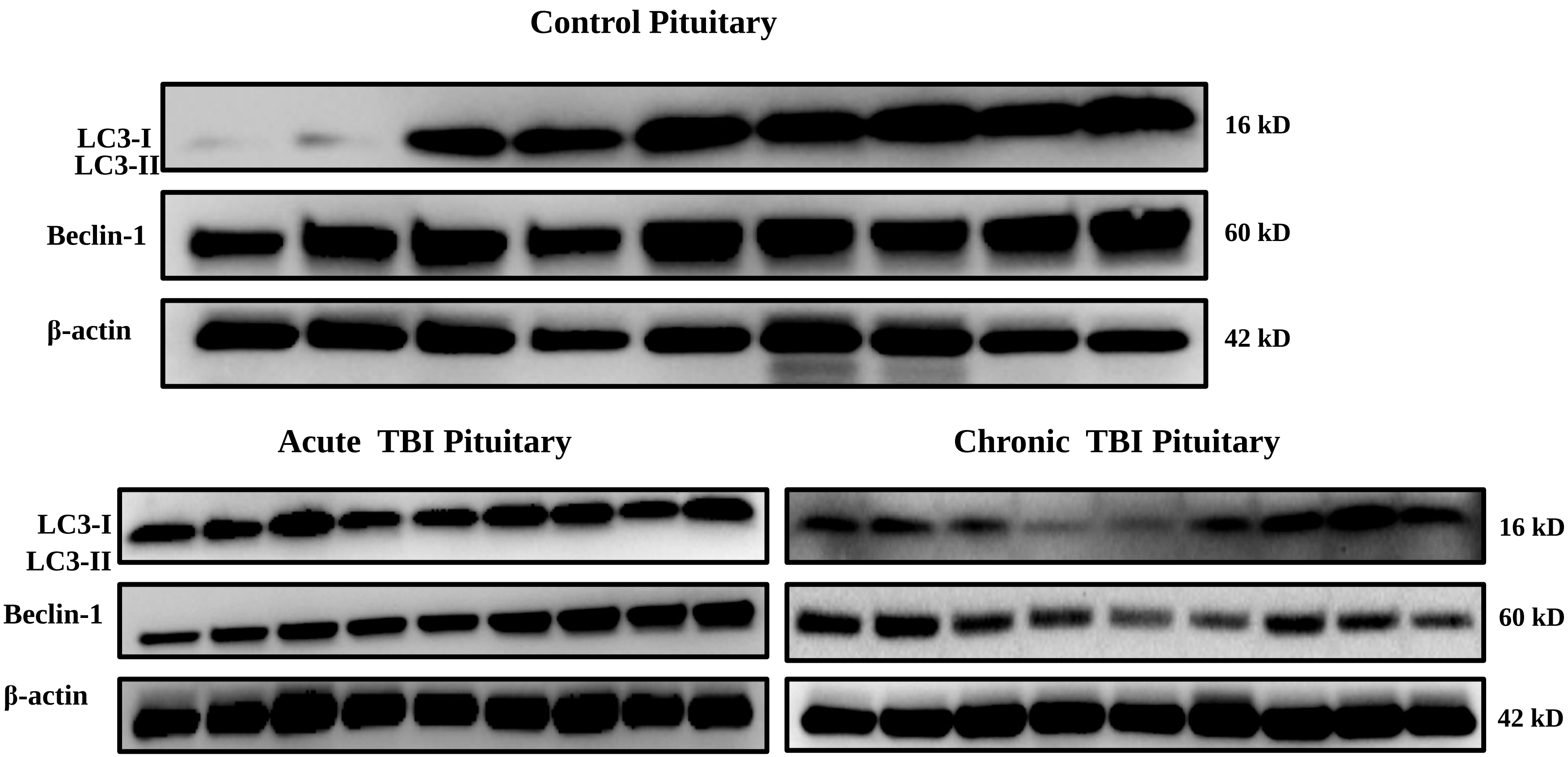

Supp. Figure S1. B. Western blot images of LC3-I and Beclin-1 in the pituitary after TBI in the acute, chronic, and control groups.

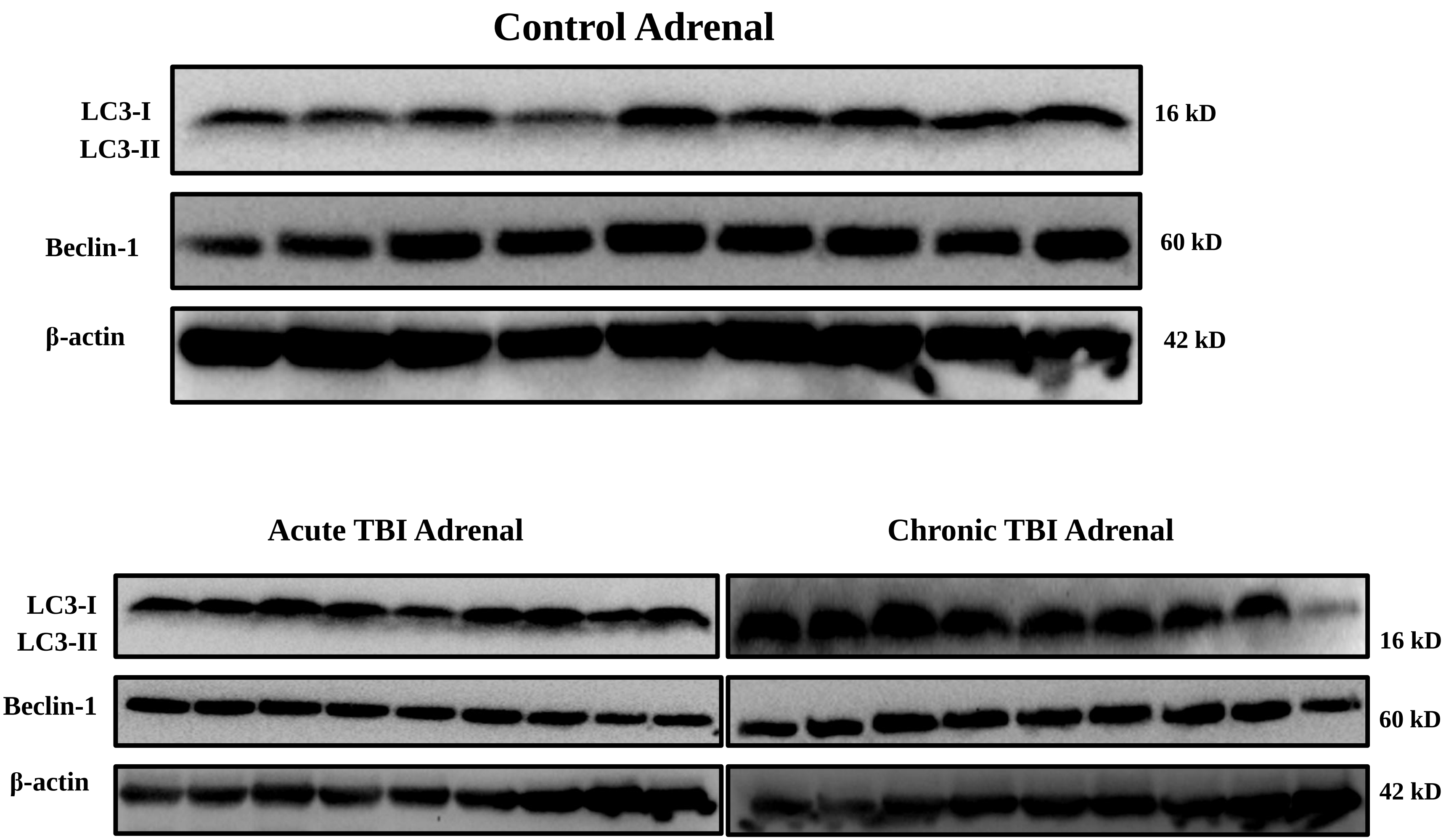

Supp. Figure S1. C. Western blot images of LC3-I and Beclin-1 in the adrenals after TBI in the acute, chronic, and control groups.
